# Dominance of Asia II 1 species of *Bemisia tabaci* in Pakistan and beyond

**DOI:** 10.1038/s41598-022-05612-1

**Published:** 2022-01-27

**Authors:** Muhammad Arslan Mahmood, Nasim Ahmed, Sonia Hussain, Sidra Tul Muntaha, Imran Amin, Shahid Mansoor

**Affiliations:** Agricultural Biotechnological Division, National Institute for Biotechnology and Genetic Engineering, Pakistan Institute of Engineering and Applied Sciences, Faisalabad, Pakistan

**Keywords:** Biological techniques, Genetics, Molecular biology

## Abstract

Globally, Whitefly (*Bemisia tabaci*) is one of the most important insect pests of crops that causes huge economical losses. The current study was designed to exclusively screen the *B. tabaci* species in the cotton field of Pakistan during 2017–2020 and have to conduct comparative analysis of *B. tabaci* species in Asia where Asia II 1 has been reported. A total of 5142 *B. tabaci* sequences of mitochondrial cytochrome oxidase 1 (mtCO1) from Asian countries were analyzed to determine the species and their distribution in the region. Our analysis over time and space showed that Asia II 1 has gradually dominated over Asia 1 in Punjab Province and over both Asia 1 and MEAM1 in Sindh Province. Asia has been divided into three regions i.e., South Asia (2524 sequences), Southeast Asia (757 sequences) and East Asia (1569 sequences) and dominance of different species of *B. tabaci* has been determined by calculating the relative percentage of each species. Interestingly, Asia II 1 has been found dominant in the neighboring region (northern zone) of India and also being dominant in its central zone. The dominance of Asia II 1 in Pakistan and northern India explains whitefly epidemic being reported in recent years.

## Introduction

Cotton (*Gossypium* spp.) is one of the most important fiber producing and oil-yielding crops. In terms of production, Pakistan is among the top 5 cotton producing countries including China, India, Brazil, and USA^1^. With the passage of time, its production decreased due to multiple biotic and abiotic factors. Among the biotic factors, insect pests are the major factor that cause losses of up to 2.5 million bales^2^. Insect pests of cotton can be divided into two major categories; chewing insects and sap-sucking insects. Chewing insects mainly consists of cotton bollworm (*Helicoverpa armigera*), spotted bollworm (*Earias insulana*), pink bollworm (*Pectinophora gossypiella*), and armyworm (*Spodoptera littoralis*) while sucking insects include whitefly (*Bemisia tabaci*), aphids (*Aphis gossypii*), jassids mealybug, and thrips (*Thrips tabaci*).

There are more than 1000 species of whiteflies^3^ among them *Bemisia tabaci* (Gennadius; Hemiptera: Aleyrodidae) is distinguished from others because of its invasiveness and fitness to the environment. It has broad host range with some host preferences. Most of the host plants of *B. tabaci* belong to the family *Cucurbitaceae, Euphorbiaceae, Malvaceae, and Solanaceae* such as cotton, tomato, eggplant, cucumber, squash, cassava, and okra, etc^4,5^. It drains the plants’ nutrients by sucking the phloem sap and also causes indirect damage by secreting honeydew which promotes fungal growth. It can transmit more than 400 plant viruses^6^ that belong to different virus genera including *Begomovirus, Crinivirus, Torradovirus,* and *Ipomovirus*. Its ability to transmit the virus in some economically important crops makes it a more devastating pest. Particularly, the complex of *Begomovirus* and *B. tabaci* is proved to be more destructive for crops in developing countries. *B. tabaci* collectively causes economic loss of billions of dollars around the world every year.

*Bemisia tabaci* is not a single species but a complex of 46 cryptic species that includes, Africa, Asia I, Asia II 1–12, Asia 1-India, Asia III, Asia IV, Asia V, Australia, Australia/Indonesia, China1-7, Indian Ocean, Ru, Middle East Asia Minor I-II (MEAM), Mediterranean (MED), MEAM K, New World 1–2. Japan 1–2, Uganda, Italy 1, and Sub Saharan Africa 1–5^7^. These species have different and overlapping biological traits. Earlier, these species were referred to as biotypes. The differentiation of these biotypes was based on the biological characteristics which primarily include the capacity to transmit viruses, variation in host range, physiological changes in host plants, capacity to produce female offspring following interspecies mating trails, capacity to disperse widely and insecticide resistance.

Biological traits were not sufficient to separate groups because of plasticity and variability of traits within and between groups. Allozymes markers (primarily esterase) were the first molecular approach to resolve the diversity within the *B. tabaci*^8,9^. Based on esterase profiling biotypes were alphabetically designated from A to T. Some other molecular markers, mt16S ribosomal DNA, *mitochondrial cytochrome oxidase 1* (mtCO1) and the nuclear ribosomal intergenic spacer 1 (ITS1) were also explored to find the genetic differences among biotypes. The mtCO1 gene is highly valuable and has been widely used for classifying haplotypes of *B. tabaci* and its close relatives. Based on threshold of 3.5% pairwise genetic divergence in mtCO1, the *B. tabaci* was resolved into complete or partial reproductively isolated groups. Lee et al.^10^ suggested that the threshold of the species boundary in the *B. tabaci* complex is required to be replaced with 4% genetic divergence. Based on mtCO1 sequences a revised nomenclature was established which tells the geographical affiliation of genetic groups. The term biotype was abandoned and genetic groups were described as cryptic species of *B. tabaci* complex.

The two highly invasive whitefly species, MEAM1 and MED originated from Middle East regions, are now invading other countries of the world^11^. In Pakistan, MEAM1 is mainly found in the southern region (Sindh province) while Asia II 1 is the most prevalent species in central and north western regions. Other than these species, Asia 1, Asia II 5, Asia II 7, and Asia II 8 have also been reported from Pakistan^12^.

The current study was designed to explore the genetic diversity of *B. tabaci* on cotton crop with mtCO1 marker in Pakistan. The second objective was to check the genetic diversity of *B. tabaci* in other Asian countries including all three major Asia regions including South Asia, South East Asia, and East Asia. Moreover, recently, Asia II 1 is also reported from Syria in Middle East region so the last objective was to check the status of different species of *B. tabaci* in Middle East countries. The findings of this study have described the current status of different species of *B. tabaci* in Asian countries. This will be helpful for the development of future management strategies to control this devastating pest in the region.

## Results

### *B. tabaci* infestation; a major constraint to cotton production in Pakistan

**Figure 1:**
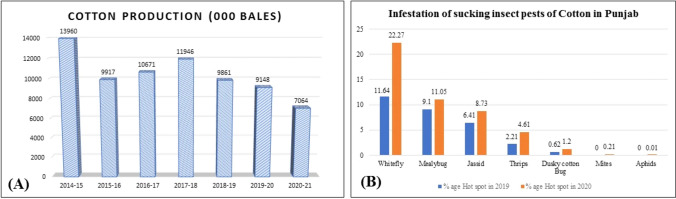
**(A)** Cotton production of Pakistan **(B)** Whitefly infestation in cotton belt of Punjab, Pakistan.

In the last few years, cotton production in Pakistan is dramatically decreased due to various reasons. The data present in Fig. 1A shows the comparison of cotton production from year 2014 to 2021. There is a marked decrease in the production of cotton. In year 2014, the cotton production was 13.9 million bales which reduced to almost half i.e., 7 million bales in 2020–2021. The data present in Fig. 1B shows 2X infestation in year 2020 in comparison of year 2019, where the infestation percentage was 11.64%.

### Identification of *B. tabaci* species from Pakistan

**Figure 2:**
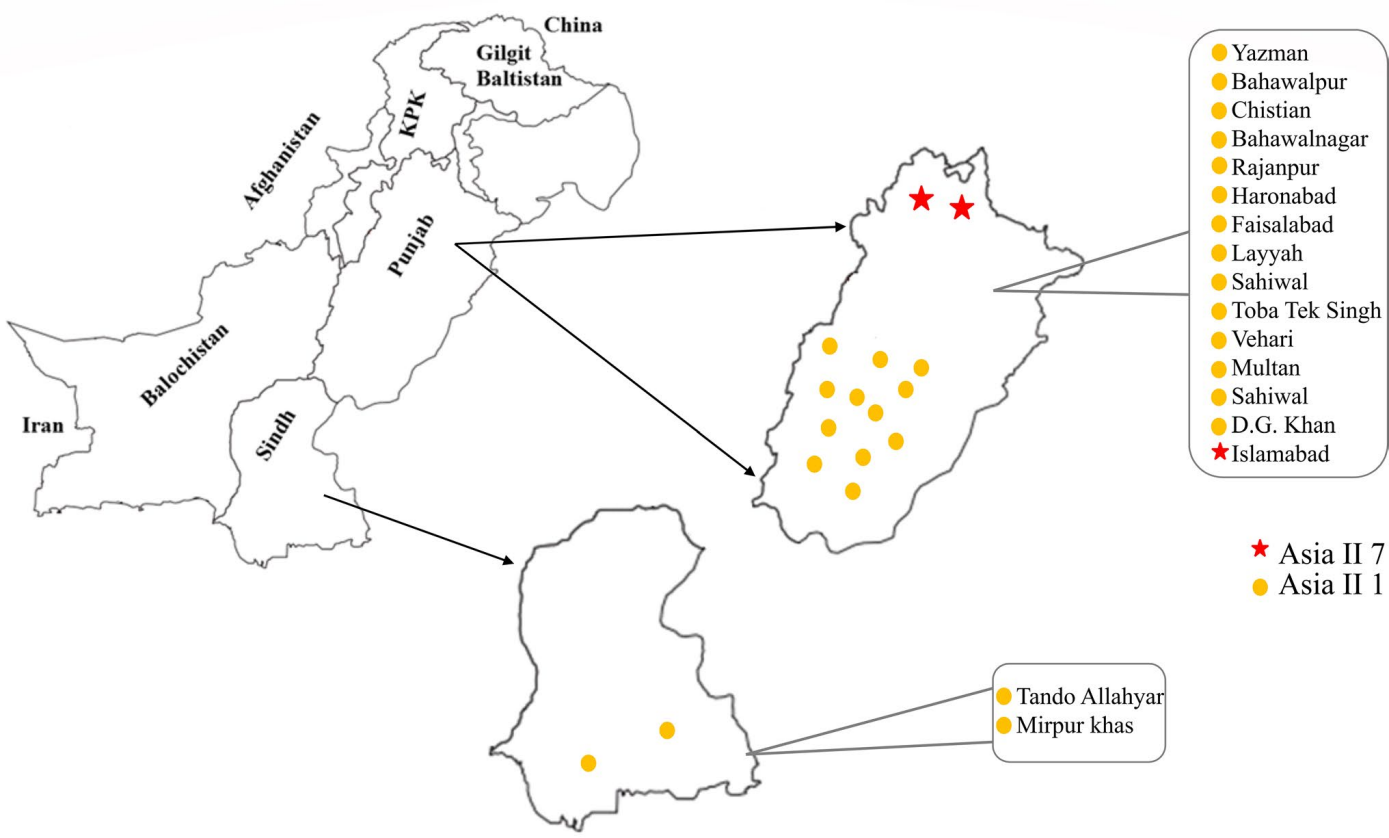
Map of Pakistan showing labelled districts, from where samples of *B. tabaci* were collected. The map was generated using CorelDRAW 12 software and edited in Paint 3D and Microsoft PowerPoint 2019.

Samples of whitefly were collected from 2017 to 2020 from different provinces (Punjab and Sindh) of the country (Fig. 2). Collectively, we identified 80/82 sequences of Asia II 1 and 2/82 sequences of Asia II 7 species from Pakistan. No other species was detected in the current study. Asia II 7 was only detected from samples collected from Islamabad; the city in northern areas that is not the major cotton growing areas. The samples detail which was collected in the current study is summarized in Table 1.

**Table 1:** Detail of *B. tabaci* sequences identified in the current study.

Sr. #	Acc. #	Species	Province	City	Latitude	Longitude	Isolate	Col. Year
1	MZ313270	Asia II 1	Punjab	Yazman	29.14467N	71.73595E	AW-3	2020
2	MZ313271	Asia II 1	Punjab	Yazman	29.146170N	71.743499E	AW-4	2020
3	MZ313272	Asia II 1	Punjab	Yazman	29.146170N	71.746760E	AW-5	2020
4	MZ313273	Asia II 1	Punjab	Yazman	29.137623N	71.756717E	AW-6	2020
5	MZ313274	Asia II 1	Punjab	Yazman	29.132226N	71.734229E	AW-7	2020
6	MZ313275	Asia II 1	Punjab	Yazman	29.129527N	71.761008E	AW-8	2020
7	MZ313276	Asia II 1	Punjab	Yazman	29.124428N	71.737662E	AW-9	2020
8	MZ313277	Asia II 1	Punjab	Yazman	29.108081N	71.738520E	AW-10	2020
9	MZ313278	Asia II 1	Punjab	Yazman	29.097132N	71.744872E	AW-11	2020
10	MZ313279	Asia II 1	Punjab	Bahawalpur	29.347898N	71.671470E	AW-13	2020
11	MZ313280	Asia II 1	Punjab	Bahawalpur	29.358711N	71.652937E	AW-14	2020
12	MZ313281	Asia II 1	Punjab	Bahawalpur	29.364974N	71.735778E	AW-15	2020
13	MZ313282	Asia II 1	Punjab	Chistian	29.797352N	72.836743E	AW-16	2020
14	MZ313283	Asia II 1	Punjab	Chistian	29.795341N	72.835606E	AW-17	2020
15	MZ313284	Asia II 1	Punjab	Chistian	29.790987N	72.834200E	AW-18	2020
16	MZ313285	Asia II 1	Punjab	Chistian	29.781153N	72.844457E	AW-19	2020
17	MZ313286	Asia II 1	Punjab	Bahawalnagar	30.028904N	73.229412E	AW-20	2020
18	MZ313287	Asia II 1	Punjab	Bahawalnagar	30.014449N	73.230711E	AW-21	2020
19	MZ313288	Asia II 1	Punjab	Bahawalnagar	30.010527N	73.229655E	AW-22	2020
20	MZ313289	Asia II 1	Punjab	Rajanpur	29.112637N	70.317262E	AW-23	2020
21	MZ313290	Asia II 1	Punjab	Rajanpur	29.114078N	70.313188E	AW-24	2020
22	MZ313291	Asia II 1	Punjab	Rajanpur	29.113994N	70.316668E	AW-25	2020
23	MZ313292	Asia II 1	Punjab	Rajanpur	29.111647N	70.309053E	AW-26	2020
24	MZ313293	Asia II 1	Punjab	Rajanpur	29.104118N	70.311337E	AW-27	2020
25	MZ313294	Asia II 1	Punjab	Haronabad	29.627958N	73.120594E	AW-28	2020
26	MZ313295	Asia II 1	Punjab	Haronabad	29.634530N	73.137314E	AW-29	2020
27	MZ313296	Asia II 1	Punjab	Haronabad	29.608393N	73.152638E	AW-30	2020
28	MZ313297	Asia II 1	Punjab	Haronabad	29.596281N	73.149364E	AW-31	2020
29	MZ313298	Asia II 1	Punjab	Haronabad	29.594717N	73.147816E	AW-32	2020
30	MZ313299	Asia II 1	Punjab	Faisalabad	31.397675N	73.026141E	AW-33	2020
31	MZ313300	Asia II 1	Punjab	Layyah	30.984232N	70.928402E	AW-36	2020
32	MZ313301	Asia II 1	Punjab	Layyah	30.979178N	70.927938E	AW-37	2020
33	MZ313302	Asia II 1	Punjab	Sahiwal	30.677757N	73.041449E	AW-39	2020
34	MZ313303	Asia II 1	Sindh	Tando Allahyar	25.472777N	68.724987E	AW-41	2020
35	MZ313304	Asia II 1	Sindh	Mirpur Khas	25.506464N	69.003288E	AW-42	2020
36	MZ313305	Asia II 7	Punjab	Islamabad	33.662970N	73.124633E	AW-44	2020
37	MZ313306	Asia II 7	Punjab	Islamabad	33.665728N	73.127134E	AW-45	2020
38	MZ313307	Asia II 1	Punjab	Islamabad	33.671428N	73.125817E	AW-46	2020
39	MZ313308	Asia II 1	Punjab	Islamabad	33.673854N	73.118686E	AW-47	2020
40	MK357328	Asia II 1	Punjab	TT Singh	30.977921N	72.466899E	STM-1	2017
41	MK357329	Asia II 1	Punjab	TT Singh	30.981035N	72.462602E	STM-2	2017
42	MK357330	Asia II 1	Punjab	TT Singh	30.972902N	72.471313E	STM-3	2017
43	MK357331	Asia II 1	Punjab	TT Singh	30.955377N	72.474908E	STM-4	2017
44	MK357332	Asia II 1	Punjab	Vehari	30.062332N	72.340713E	STM-5	2017
45	MK357333	Asia II 1	Punjab	Vehari	30.062413N	72.332172E	STM-6	2017
46	MK357334	Asia II 1	Punjab	Vehari	30.060854N	72.329262E	STM-7	2017
47	MK357335	Asia II 1	Punjab	Vehari	30.059187N	72.325681E	STM-8	2017
48	MK357336	Asia II 1	Punjab	Multan	30.167339N	71.572929E	STM-9	2017
49	MK357337	Asia II 1	Punjab	Multan	30.166079N	71.570058E	STM-13	2017
50	MK357338	Asia II 1	Punjab	Multan	30.259145N	71.541788E	STM-14	2017
51	MK357339	Asia II 1	Punjab	Multan	30.263070N	71.436380E	STM-15	2017
52	MK357340	Asia II 1	Punjab	Sahiwal	30.619366N	73.079417E	STM-16	2017
53	MK357341	Asia II 1	Punjab	Sahiwal	30.630009N	73.069540E	STM-17	2017
54	MK357342	Asia II 1	Punjab	Sahiwal	30.612582N	73.069595E	STM-18	2017
55	MK357343	Asia II 1	Punjab	Sahiwal	30.608004N	73.099384E	STM-19	2017
56	MK357344	Asia II 1	Punjab	D.G. Khan	30.048206N	70.677084E	STM-20	2017
57	MK357345	Asia II 1	Punjab	D.G. Khan	30.044698N	70.676435E	STM-21	2017
58	MK357346	Asia II 1	Punjab	D.G. Khan	30.045584N	70.670634E	STM-22	2017
59	MK357347	Asia II 1	Punjab	D.G. Khan	30.050709N	70.669943E	STM-25	2017
60	MK357348	Asia II 1	Punjab	TT Singh	30.959737N	72.483569E	STM-26	2018
61	MK357349	Asia II 1	Punjab	TT Singh	30.958983N	72.483548E	STM-28	2018
62	MK357350	Asia II 1	Punjab	TT Singh	30.956623N	72.484775E	STM-29	2018
63	MK357351	Asia II 1	Punjab	TT Singh	30.952657N	72.482393E	STM-30	2018
64	MK357352	Asia II 1	Punjab	Vehari	30.033080N	72.362021E	STM-33	2018
65	MK357353	Asia II 1	Punjab	Vehari	30.033674N	72.364478E	STM-34	2018
66	MK357354	Asia II 1	Punjab	Vehari	30.033785N	72.366474E	STM-35	2018
67	MK357355	Asia II 1	Punjab	Vehari	30.032717N	72.367000E	STM-36	2018
68	MK357356	Asia II 1	Punjab	Vehari	30.028398N	72.367730E	STM-37	2018
69	MK357357	Asia II 1	Punjab	Multan	30.130419N	71.571077E	STM-38	2018
70	MK357358	Asia II 1	Punjab	Multan	30.129276N	71.574950E	STM-39	2018
71	MK357359	Asia II 1	Punjab	Multan	30.128144N	71.573845E	STM-40	2018
72	MK357360	Asia II 1	Punjab	Multan	30.202989N	71.596566E	STM-41	2018
73	MK357361	Asia II 1	Punjab	Multan	30.270839N	71.592235E	STM-42	2018
74	MK357362	Asia II 1	Punjab	Sahiwal	30.651294N	73.129210E	STM-43	2018
75	MK357363	Asia II 1	Punjab	Sahiwal	30.649254N	73.127086E	STM-44	2018
76	MK357364	Asia II 1	Punjab	Sahiwal	30.649965N	73.123030E	STM-45	2018
77	MK357365	Asia II 1	Punjab	Sahiwal	30.637863N	73.108079E	STM-46	2018
78	MK357366	Asia II 1	Punjab	Sahiwal	30.637992N	73.102178E	STM-47	2018
79	MK357367	Asia II 1	Punjab	D.G. Khan	30.050365N	70.669906E	STM-48	2018
80	MK357368	Asia II 1	Punjab	D.G. Khan	30.048878N	70.673560E	STM-49	2018
81	MK357369	Asia II 1	Punjab	D.G. Khan	30.046821N	70.670049E	STM-50	2018
82	MK357370	Asia II 1	Punjab	D.G. Khan	30.014736N	70.629576E	STM-51	2018

### Phylogenetic analysis of mtCO1 sequences of *B. tabaci*

**Figure 3:**
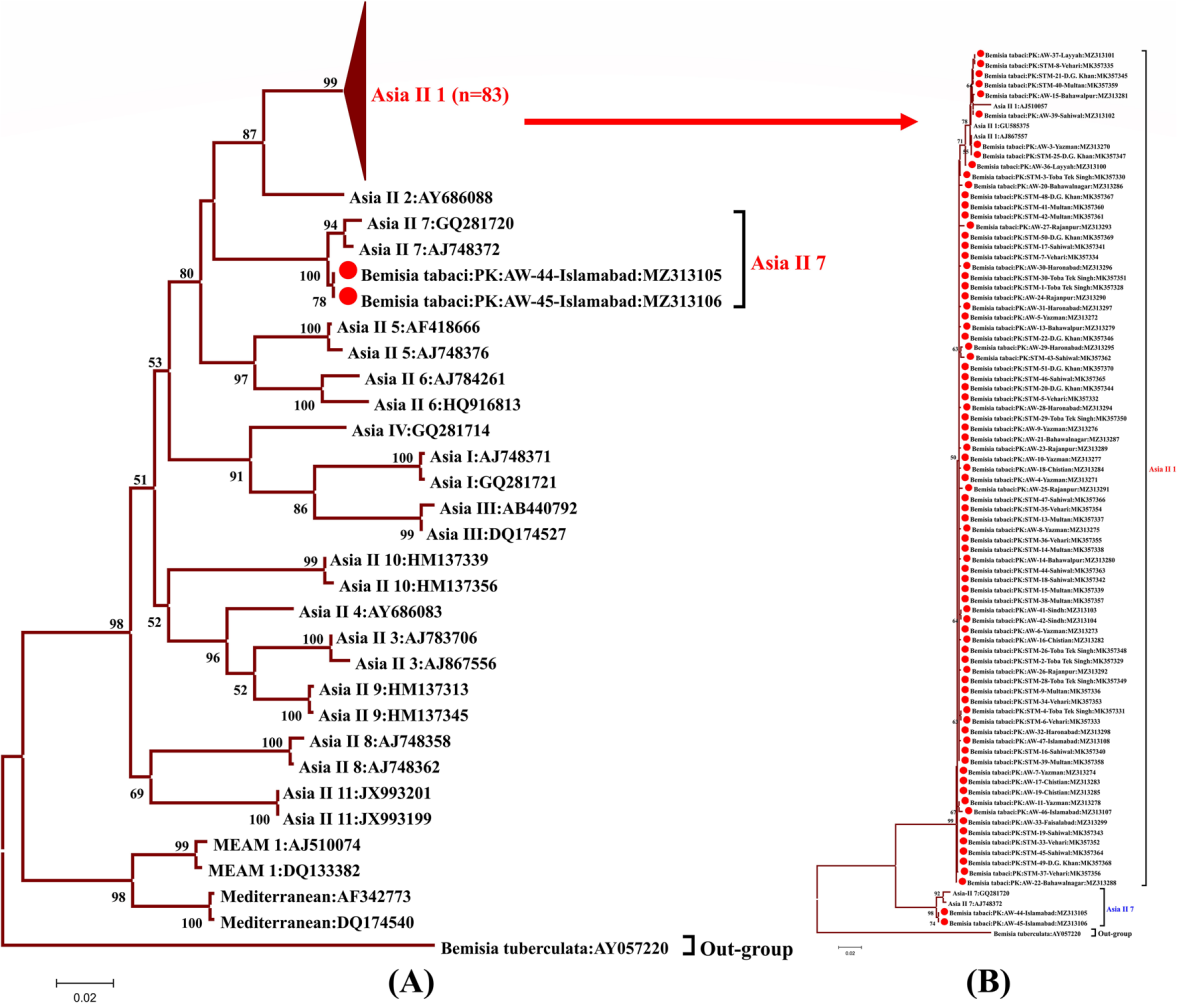
**(A)** A neighbor-joining phylogenetic tree was reconstructed to infer the relationship of mtCO1 sequences identified in the current study with the already known species of *B. tabaci*. Tree was rooted on the sequence of *Bemisia tuberculata* (AY057220) species and a total of 113 sequences were used in this analysis including 82 sequences identified in the current study and other reference sequences of some other species. The multiple sequences alignment was carried out by MUSCLE program in MEGA7 software^32^. Bootstrap method was used with 1000 replications and the percentage bootstrap value (greater than 50%) is shown on each breach. The 80 sequences identified in the current study made the group with Asia II 1 reference sequences (n = 3) and this group is collapsed at the top of the tree showing Asia II 1 (n = 82) whereas two sequences identified in the current study made the clade with Asia II 7 reference sequences. The isolates of the current study are labelled with red circle in the start of their mentioned name in the tree. **(B)** A neighbor-joining phylogenetic tree was reconstructed by using the current sequences and the reference sequences of only species identified in the current study and an out-group sequence mentioned above. This was included to show the relations among the sequences of the current study that was collapsed in **(A)**.

Phylogenetic dendrogram was constructed using 82 nucleotide mtCO1 sequences of *B. tabaci* and *B. tuberculata* as an outgroup. Tree analysis showed the presence of two species of *B. tabaci* in the current study. No other species of *B. tabaci* was identified in the current study. The samples (MZ313303, MZ313304) collected from the Sindh cotton growing areas, were also showing the similarity with Asia II 1. The neighbor-joining phylogenetic tree was reconstructed to infer the relationship of the sequences identified in the current study is shown in Fig. 3A and B.

### Distribution of *B. tabaci* in Pakistan at the province level

**Figure 4:**
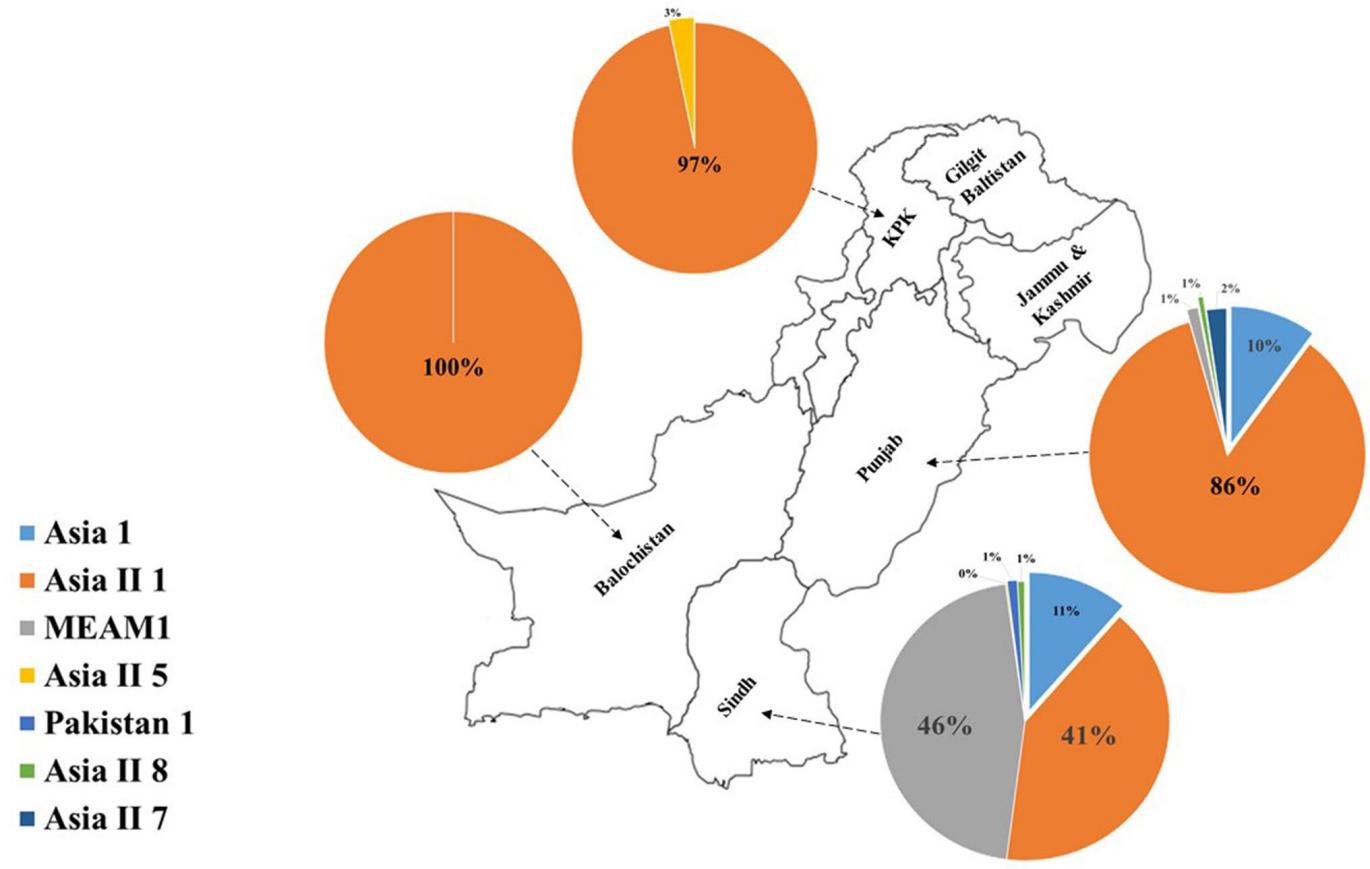
Distribution of *B. tabaci* in Pakistan at province level. The relative % age of each species is shown in pie diagram against each province. The map was generated using CorelDRAW 12 software and edited in Paint 3D and Microsoft PowerPoint 2019.

In total 1096 sequences were so far reported from Pakistan after removing the 5’ mtCOI gene sequences and also those sequences that contain somehow wrong information (actually they belong to some other species of *Bemisia* genus). These 1096 sequences were phylogenetically analyzed to determine their species. There were eight species presently named as Asia II 1, MEAM1, Asia II 7, Asia 1, Asia II 5, Asia II 8, Pakistan and Pakistan 1. A single sequence (KJ709461) named as “Pakistan” species was previously reported from Islamabad^13^ whereas a single sequence (GU585374) was named “Pakistan-1” during the current analysis and according to GenBank submission data it was reported from Sindh province of Pakistan. The relative percentage of each species was determined and shown in the pie diagram against each province in Fig. 4. The Asia II 1 species showed the highest relative % in Balochistan, KPK and Punjab provinces where MEAM1 showed the highest relative % in Sindh province followed by Asia II 1 species.

**Figure 5:**
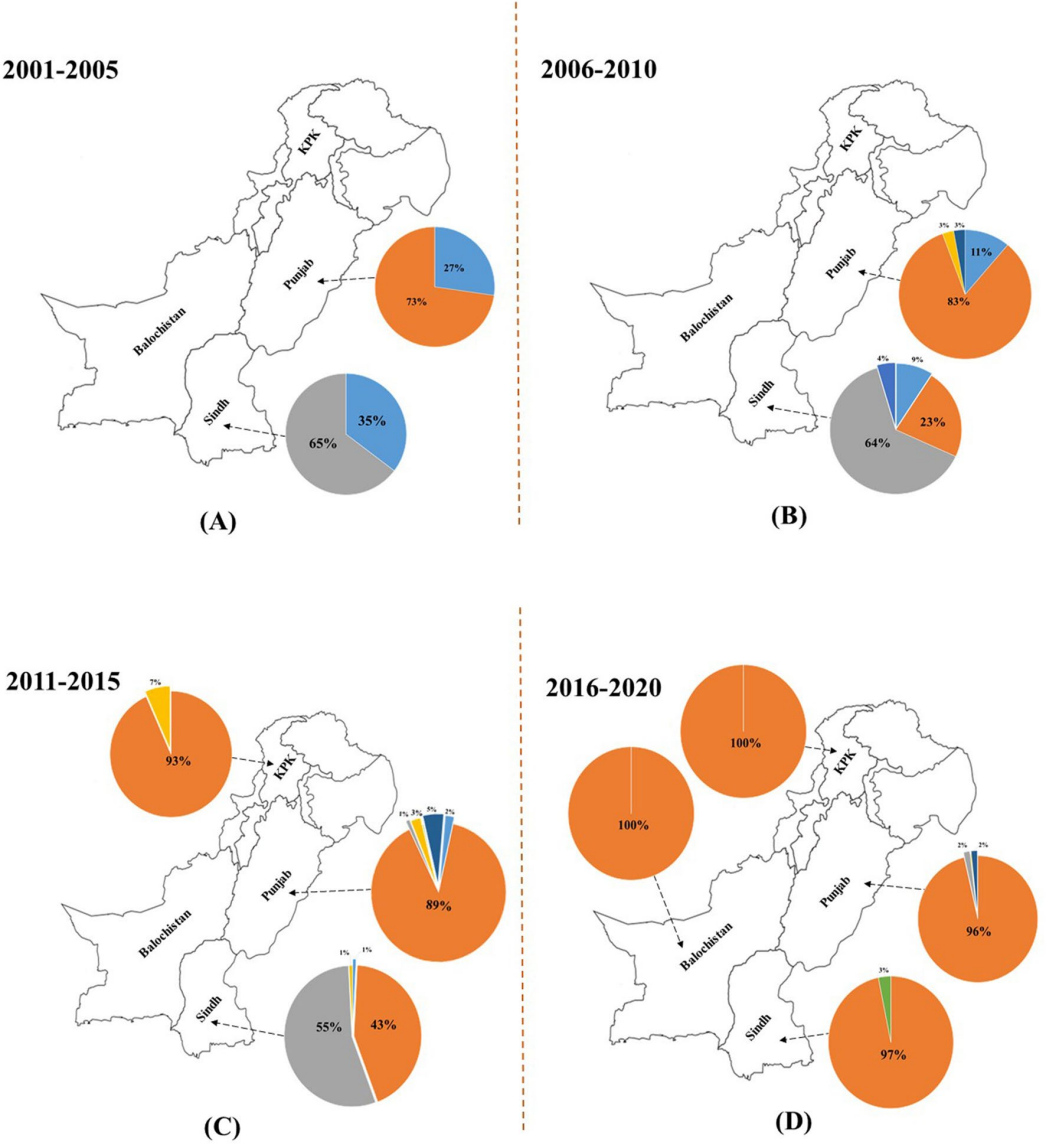
Relative % age of each species was determined with the timeline (an interval of five years). The relative % age of different species of *B. tabaci* reported from 2001 to 2005, 2006–2010, 2011–2015 and 2016–2020. The map was generated using CorelDRAW 12 software and edited in Paint 3D and Microsoft PowerPoint 2019.

To further elaborate the situation, relative % of each species was determined with the timeline (an interval of 5 years). The relative % of different species of *B. tabaci* reported from 2001 to 2005, 2006–2010, 2011–2015 and 2016–2020, shown in Fig. 5A–D.

### Distribution of *B. tabaci* from other countries in South Asia

**Figure 6:**
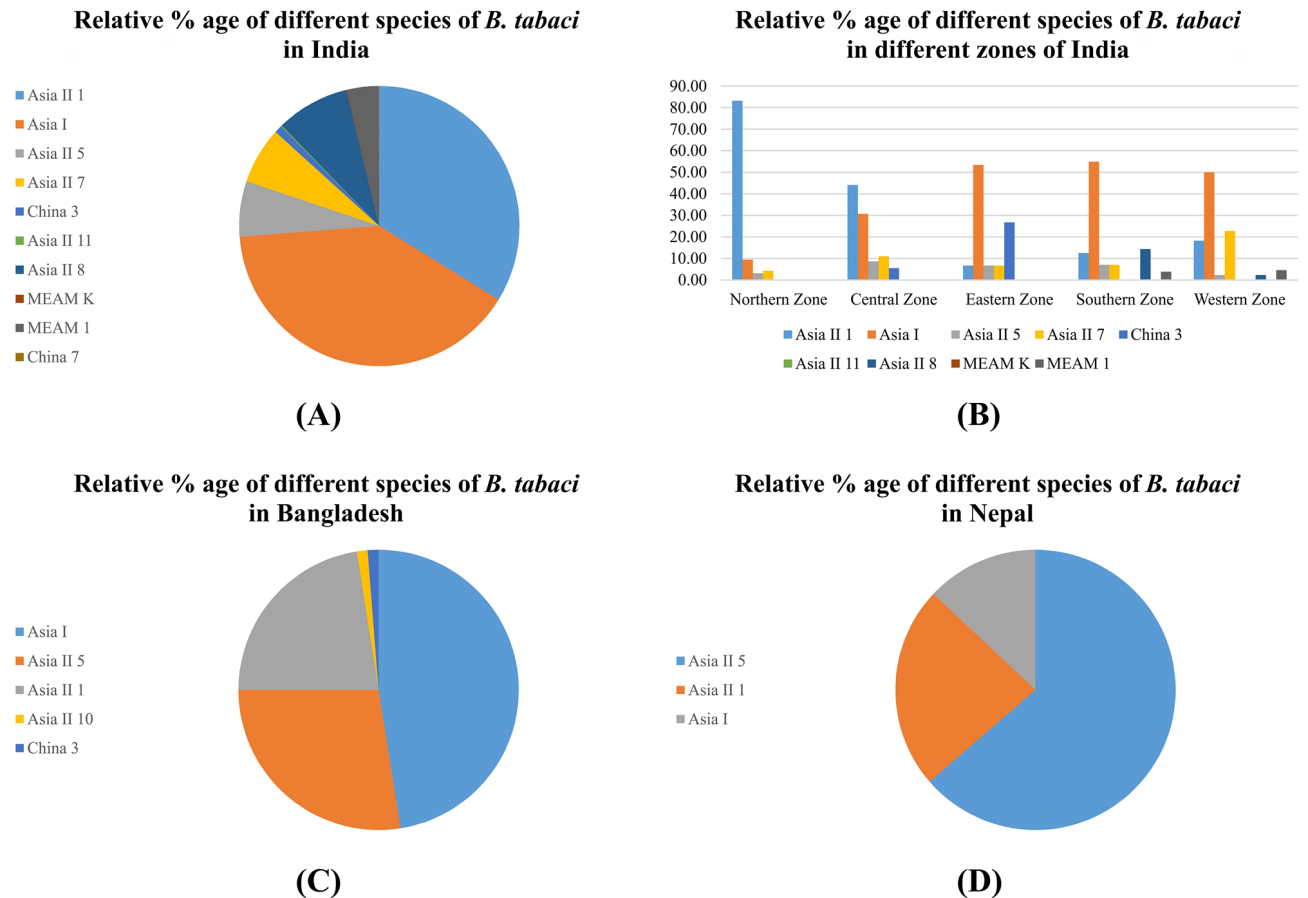
Distribution of different species of *B. tabaci* in other countries of South Asia. **(A)** The relative % of different species of *B. tabaci* so far reported from India. **(B)** The relative % of different species of *B. tabaci* from different zones of India. **(C)** The relative % of different species of *B. tabaci* from Bangladesh and **(D)** The relative % of different species of *B. tabaci* from Nepal.

In total 1166 sequences were so far reported from India after removing the 5′ mtCOI gene sequences and those sequences which contain incomplete information (they were belonging to some other species of *Bemisia* genus). These 1166 sequences were analyzed to determine their species. The data present in Fig. 6A shows the relative % age of *B. tabaci* species in India where Asia 1 is dominant over the Asia II 1, Asia II 5, Asia II 7, and Asia II 8. As India is very large country as compared to Pakistan so its zones were used for the comparison of different species in these zones. Overall, India is divided into 6 zones, out of these, *B. tabaci* is reported from five zones shown in Fig. 6B with the relative % age of each species reported. Like Pakistan, Asia II 1 is dominant in its neighboring zone of India i.e., Northern zone. Interestingly it is also dominant in Central zone of India.

The other countries of South Asia where *B. tabaci* has been reported are Bangladesh, Nepal and Afghanistan. The relative % of each species from Bangladesh and Nepal is shown in Fig. 6C and **(D)** respectively. From Afghanistan only two species i.e., Asia 1 and Asia II 5 with few sequences were reported (data not shown here). Moreover, a list of all sequences analyzed is provided in the supplementary file S2, Table S1.

### Distribution and genetic variation of *B. tabaci* in South East Asian countries

**Figure 7:**
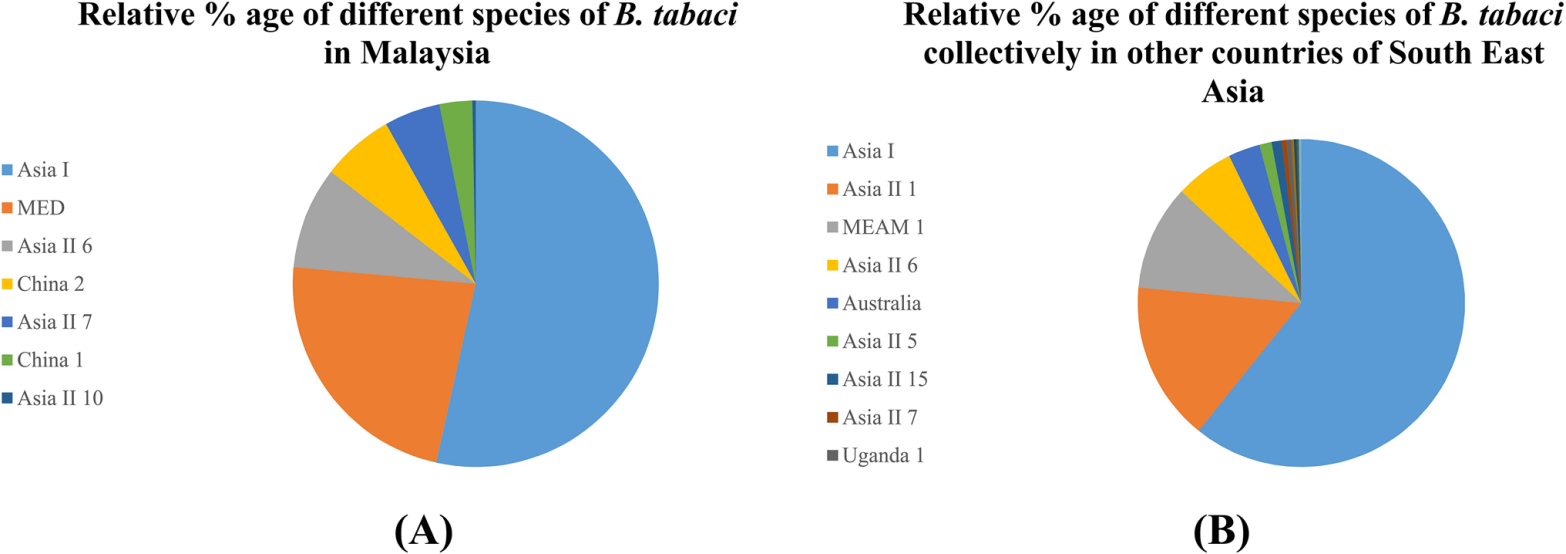
*Bemisia tabaci* species distribution in South East Asia. **(A)** The relative % of each species of *B. tabaci* so far reported from Malaysia, **(B)** shows the relative % of different species of *B. tabaci* collectively reported from other countries in South East Asia.

Different species of *B. tabaci* have been reported from eight countries of South East Asia including Indonesia, Myanmar, Singapore, Cambodia, Vietnam, Philippines, Thailand and Malaysia. The largest number of sequences have been reported from Malaysia where Asia 1 species have the largest number of sequences so far reported followed by MED, Asia II 6, China 2, Asia II 7, China 1, and Asia II 10. The relative % these species reported from Malaysia have been shown in Fig. 7A. Interestingly, Asia II 1 is still not reported from Malaysia but it has been reported from some other countries in the Southeast Asian region like Cambodia, Thailand and Vietnam. The overall combined trend of distribution of different species of *B. tabaci* in these countries is shown in Fig. 7B. Moreover, a list of all sequences analyzed is provided in the supplementary file S2, Table S2.

### Distribution and genetic variation of *B. tabaci* in East Asia and Middle East countries

**Figure 8:**
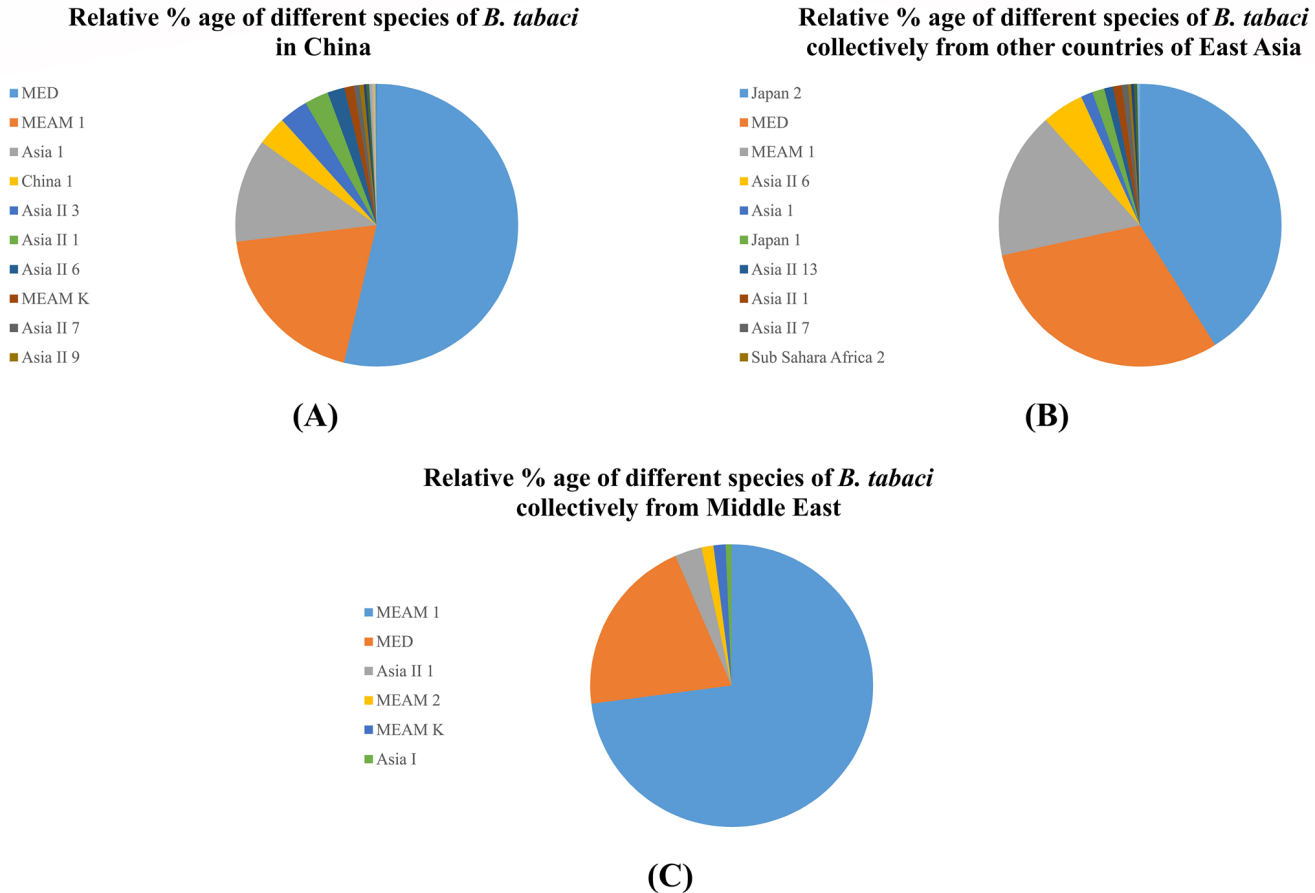
*Bemisia tabaci* species distribution in East Asian countries. **(A)** The relative % of different species of *B. tabaci* so far reported from China, **(B)** The relative % of different species of *B. tabaci* collectively from other countries in East Asian region, **(C)** The relative % of different species of *B. tabaci* from Middle East countries.

In East Asia, the largest number of sequences of *B. tabaci* have been reported from China. So, the analysis of data from China is separately described in Fig. 8A. MED is dominant in China followed by MEAM1, Asia 1 and others. The other countries of East Asia region where *B. tabaci* has been reported are Japan, South Korea and Taiwan. The relative % of different species of *B. tabaci* collectively from the other countries in East Asia region is shown in Fig. 8B. The relative % of different species of *B. tabaci* collectively from countries in Middle East is shown in Fig. 8C. The list of sequences of East Asia are provided in supplementary file S2, Table S3 whereas sequences of Middle East are provided in supplementary file S2, Table S4.

### Prevalence of Asia II 1 species in Asia

**Figure 9:**
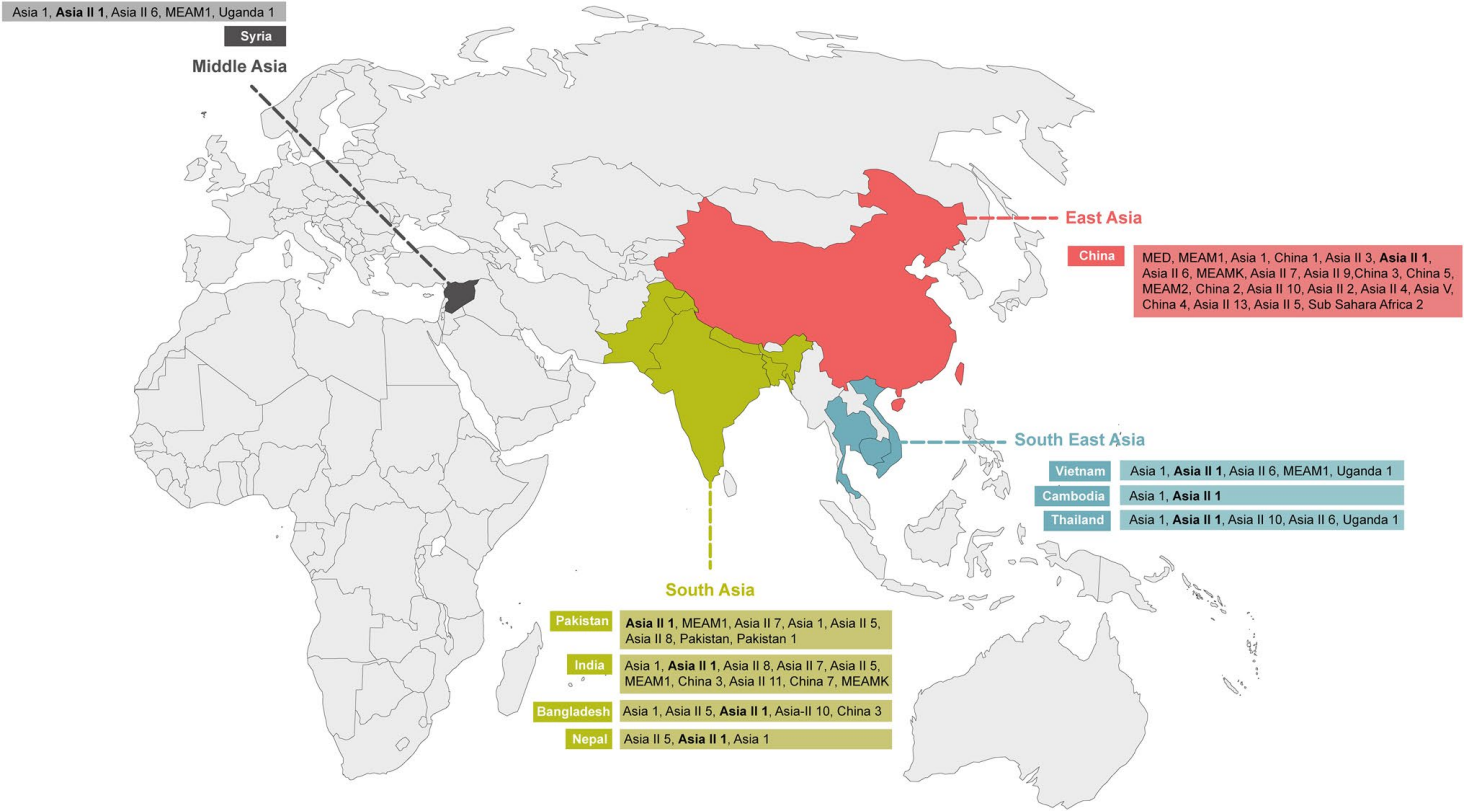
Geographical distribution of *B. tabaci* species across the world. The presence of different species identified from different geographical locations is indicated in multiple colors. **Asia II 1** identified from different countries is in bold font. The world map was collected from https://simplemaps.com and modified using CorelDRAW 12.

Asia II 1 species has been confirmed from 10 countries including Pakistan, India, Bangladesh, Nepal, Cambodia, Vietnam, Thailand, Taiwan, China and Syria. The list of species so far reported from each country has been summarized in supplementary file S1, Table 1. The map of the countries where Asia II 1 species has been reported is shown in Fig. 9.

## Conclusion and future outlook

In the present study, it has been found that *B. tabaci* species, Asia II 1 is dominant in Pakistan and its spread in comparison with other species has been determined from other Asian countries. This reports the overall situation of different species of *B. tabaci* in the region. In the future, similar study can be designed to determine the *B. tabaci* species from the remaining parts of the world. That will be helpful to understand the overall global situation of different *B. tabaci* species. Secondly, there is a need to explore the tripartite interactions at the molecular level to deeply understand the compatible interaction of specific viruses with vectors and hosts. This deeper understanding will be help break the compatible interaction and will put a way forward to the development of resistant cultivars.

## Discussion

In agriculture, species invasion is among the most important factors which drives the changes in natural equilibria and also it has been a major threat to crop production. Insect has the ability to adapt according to the environmental changes and such changes have often been due to genetic changes^14^. *B. tabaci* has become the paradigm for both genetic variability and invasiveness in various countries worldwide. The present study is significant because it provides the overall situation about the *B. tabaci* populations belonging to different species in the Asian countries of the world. The current situation of *B. tabaci* species in Pakistan is revealed by this study where the dominance of Asia II 1 over the Asia 1, MEAM1 and other species found in Pakistan has been documented. The available sequences (till 1st June 2021) of mtCO1 gene of *B. tabaci* were analyzed to determine their species in these regions. However, this conclusion has been taken based on the available sequences of mtCO1 gene of *B. tabaci* that may have bias in collecting and submitting of the sequence. Country-wise data is provided with species and years of reporting that shows the overall situation of *B. tabaci* genetic variation in these countries.

In Pakistan, we found the dominance of Asia II 1 in major cotton growing regions whereas in its north central region (Islamabad), Asia II 7 was also identified^15^. One interesting finding in the current analysis is that Asia 1 has disappeared since 2012 from the country. This shows the dominance of Asia II 1 over Asia 1 in Pakistan over the time. In India, Asia 1 is the most dominant species followed by Asia II 1 and others^7,16^. Recently, in a broader survey in India it has been found that Asia II 1 is dominant in its northern region that shows a situation similar to that of Pakistan. The major reason behind this dominance is probably the excessive use of insecticides and the development of insecticide resistance in Asia II 1 species. Due to the extensive use of insecticide, rapid development of resistance has been reported in Asia II 1 and Asia 1 species of *B. tabaci*^17^. Insecticides have been considered the mainstay in controlling the *B. tabaci* in agricultural production systems. In the late 1970s and 1980s, pyrethroids insecticides replaced the organophosphates (OPs) and organochlorine insecticides. But in the late 1990s these were also replaced by neonicotinoids and other compounds. Excessive use of the same active ingredient and increased use of insecticides within a given cropping system has led to the insecticides resistance development in *B. tabaci* against the OPs and pyrethroids^18,19^. For example, Naveen (2016) described that Asia 1 and Asia II 1 have evolved resistance against organophosphates (Chlorpyrifos, etc.) and pyrethroids (Deltamethrin, etc.) while Asia II 7 is still susceptible against these insecticides^17^. The mechanism underlying insecticides resistance has been reported in a recent study that described the whole genome sequence of Asia II 1 from Pakistan. They stated that Asia II 1 have 1294 genes with high impact variants including 14 genes have involved in insecticides resistance^17^. In other south Asian countries like Bangladesh and Nepal, they have a similar situation like India where Asia 1 and Asia II 5 acts as a dominant species followed by Asia II 1 respectively resulting in the dominance of Asia II 1 in other countries. This seems Asia II 1 is being dominant in other regions of the world.

In South East Asia, the largest number of sequences have been reported from Malaysia, where Asia 1 is dominant followed by MED and others. Interestingly, there is still no report of Asia II 1 from Malaysia whereas it has been found in some other countries in South East Asia like Cambodia, Vietnam and Thailand. In East Asia countries, the largest number of sequences have been reported from China where MED is dominant followed by MEAM1 and ~ 19 species have been found in China. Interestingly, Asia II 1 was recently reported from China where CLCuD is also recently introduced. This clearly shows the relationship of different species with specific begomoviruses diseases in a region. Japan 2 is dominant in other countries like Japan and South Korea whereas MED and MEAM1 are dominant in other countries of East Asia region. In the Middle East region MED and MEAM1 are found dominant in different countries where Asia II 1 is only reported from Syria in the region.

Why Asia II 1 became dominant in Pakistan especially in cotton regions?? There are few gestures and hypothesis by the previous reports from Pakistan. Previously, it has been reported that Asia II 1 is the vector of CLCuD causing begomoviruses/satellites in Punjab, Pakistan where its 1st and 2nd epidemics occurred and this disease has spread to Sindh and also reported from northwestern India. Recently, in a broad survey the 3rd epidemic of CLCuD has been reported from Pakistan and distinct disease complex is found associated with this disease^20,21^. The dominant strain was cotton leaf curl Multan virus (CLCuMuV-Raj) Rajasthan strain and same has been reported from India since 2015^22^. Interestingly, during a nation-wide vector-based survey of begomoviruses in Pakistan it has been found that CLCuMuV-Raj strain is the dominant strain in whitefly found from cotton regions in the country and dominant species was Asia II 1^23^ than shows the co-evolution of the tripartite (host-vector-virus) in the region.

Updated record of population surge and diversity of *B. tabaci* species in a region is critical for developing effective approaches for whitefly control and to prevent the spread of invasive species. This study reports the two species Asia II 1 and Asia II 7 with mainly predominance of the Asia II 1 on cotton crop in Punjab and Sindh province of Pakistan.

Asia II 1 has been remained dominant in the central region (Punjab where CLCuD is endemic) and MEAM1 have been predominantly found in the southern region (Sindh where TYLCD is endemic) for more than three decades. Asia II 1 and MEAM1 have been prevalent in the overlapping regions in Pakistan for a long time but even then, the MEAM1 could not succeed to displace indigenous species Asia II 1, as has been the global trend. There is persistence of Asia II 1 in the neighboring regions of Pakistan as well as in the northern and central regions of India as in the data shown in Fig. 6A. A recent report from India also reported the Asia II 1 abundance in the northern and central regions of the country^7^. These are basically the cotton growing areas of Pakistan and India. Ahmed et al.^24^ directly compare the host suitability between the Asia II 1 and MEAM1 performed similarly across all hosts but its longevity and fecundity is high on tomato plants while Asia II 1 survived best on the cotton plants. Ahmed et al.^25^ reported first time the association of CLCuD incidence with the identity and abundance of *B. tabaci* species and found out the high disease incidence with an abundance of Asia II 1. Then Pan et al.^26^ directly compared the transmission efficiency of CLCuMuV in four cryptic species, two native species (Asia 1 and Asia II 1) and two invasive species (MEAM1 and MED) and reported that CLCuMuV is the most efficiently transmitted by the Asia II 1. So, might be agroecological niches which is the result of specific tripartite interaction (host, virus, and vector) is more suitable for the persistence abundance of Asia II 1 in this region.

In this data, Asia 1 is not found while it has been previously reported from both Punjab and Sindh^13,25^. In 2017, updated data was only found in the Sindh region by Masood et al.^12^. But in few latest reports of the genetic diversity in Pakistan, Asia 1 is not found^15,23^. These reports align with the result of this study.

Because of the advancement of the high throughput a cost-effective technologies, instead of relying on a single mitochondrial gene, scientists used whole genome wide and integrated approach to accurately species identification of the *B. tabaci*^27^. Whole genome data of MEAM1, MED, and Asia II 1 have been available for comparative and evolutionary genomic studies^11,28,29^. These studies lay the foundation for the pan genomics studies which have the potential to explain the difference between invasive and native dominance of *B. tabaci*.

## Materials and methods

### Scenario of cotton production and sap-sucking insect infestation

The cotton production data was collected from the major cotton growing areas of Pakistan. The information was extracted from Pakistan Economic Survey, 2020–2021 (https://www.finance.gov.pk/survey/chapters_21/02-Agriculture). Similarly, all the sap sucking insect infestation data was collected from the surveys available on the website of Pest Warning and Quality Control of Pesticides (PWQC), Government of Punjab, Pakistan (http://pestwarning.agripunjab.gov.pk/sucking-insect-pests). This data in PWQC is made available after pest scouting where pest scouting teams survey the cotton growing areas of Punjab on regular basis (40–50 random fields/week). The areas with 100 percent pest infestation were termed as hotspots.

### Collection of whitefly samples

Adult whiteflies (*B. tabaci*) were collected from major cotton growing areas from 17 different geographical locations of Punjab, and Sindh province of Pakistan during the year 2017–2020. During the survey, the insect samples were collected randomly (in a zig-zag pattern) from the underside of the leaves using a handheld aspirator. About 20 adult whiteflies were collected from each locality and were kept in 95% ethanol and stored at – 20 °C for further experiments. The survey covered 14 districts of Punjab and 2 districts of Sindh province of Pakistan.

### DNA extraction

Genomic DNA was extracted from 82 individual adult whiteflies. The extraction of DNA was performed using the method described by Zhang (1998) with the following manipulations^30^. Adult whitefly was blotted on filter paper to absorb water and grinded in 300 µL CTAB buffer (100 mM Tris–HCl, 20 mM EDTA, and 1.4 M NaCl consisting of 0.2% β-mercaptoethanol). This mixture after grinding was taken in a microfuge tube and incubated for 15 min at 65 °C, and then one volume of chloroform was added and mixed it manually. The mixture placed in a centrifuge and spun at 13,000 rpm for 4 min and supernatant was collected. One volume of isopropanol was added and content was incubated for 30 min. The mixture was centrifuged at 12,000 rpm for 10 min and remove the supernatant. The remained pellet was washed with 70% ethanol twice. The pellet was dried at room temperature and dissolved in nuclease-free water (Thermo Fisher Scientific, Wilmington, USA). The quality and concentration of DNA was checked by using Nanodrop 2000/2000c spectrophotometer (Thermo Fisher Scientific, Wilmington, USA) and then was stored at − 20 °C for downstream processing.

### Molecular analyses of *B. tabaci* by mtCO1

The whitefly 3′-mtCO1 gene fragment (approximately 850 bases) can be amplified by polymerase chain reaction (PCR) by universal primers^31^. PCR was carried out in a total volume of 20 µL reaction tube containing 10 µL DreamTaq Green PCR Master Mix (Thermo Fischer Scientific), 0.5 pmol µL^−1^ of each primer (forward/reverse), 2 µL DNA (20 ng µL^−1^ whitefly DNA), and double-distilled water. This reaction was set up in the PCR machine (Bio-Rad, USA). The PCR conditions comprised of initial denaturation for 5 min at 94 °C, followed by 35 cycles of 94 °C for 30 s, 52 °C annealing for 1 min, 72 °C for 1 min, with a final extension of 10 min at 72 °C. The DNA was analyzed by using gel documentation system (Bio-Rad, USA). Successfully amplified products were excised and purified using Thermo GeneJET gel extraction kit (Thermo Fisher Scientific).

### Cloning, sequencing, and data analyses

The purified products were directly ligated in PTZ57R/T plasmid vector (Thermo Fischer Scientific) and transformed in competent cells of *Escherichia coli* (TOP10 strain). Confirmed clones were selected and the sequence was determined by automated bi-directional, Sanger sequencing (Genomics and Bioinformatics Research Unit, USDA-ARS Stoneville, MS) using 3730XL ABI sequencer (Applied Biosystems, USA). The sequence reads of each clone were assembled by SeqMan in DNASTAR (Madison, WI) and a consensus sequence was saved and analyzed. First, each complete sequence was searched online by BLASTn (Basic Local Alignment Searching Tool (nucleotides) on NCBI database to identify similar sequences. For the identification of species of *B. tabaci* all the sequences identified in the current study were compared with the reference sequences of the known species of *B. tabaci*. For phylogenetic tree analysis, all these sequences were first aligned with the sequences of the reference isolates by MUSCLE in MEGA7 software^32^. A Neighbor-joining phylogenetic tree was reconstructed using all these sequences to infer the relationship of the current isolates with the previously known species.

### The retrieving of mtCO1 sequences of *B. tabaci*

The available data of mtCO1 sequences of *B. tabaci* on GenBank was retrieved on 1st June 2021. A total 12,198 sequences are available under the organism’s name, *Bemisia tabaci* with mtCO1 gene. A major list was prepared in excel and filtered for the desired information. There were some isolates in which country name was not mentioned by the sequence submitter, so for these putative sequences, submitter’s country name was considered. Similarly, for many sequences sample collection date was missing so for that used the submission date and differentiated these dates (by bold for submission date used instead of actual collection date). For each country, sequences were retrieved based on the accession number against the name of the country in the list. The total final number of sequences from Asia used in the current study were 5142 isolates. For Pakistan, the reporting province name of each sequence was retrieved whereas, for India, zone names were also retrieved for 698 sequences.

### Sequence analysis from the countries of Asia

We first filtered the data based on the country names for example for Pakistan, isolated all the sequences reported from Pakistan. Then we did their phylogenetic tree analysis to identify their species. Similarly, for other countries, their isolates were separated and analyses for the identification of their species were done. In the current study, Asian countries from three regions; South Asia, East Asia and Southeast Asia were checked for the *B. tabaci* presence and its species were determined for each isolate from these countries. Moreover, the countries of Middle East were also checked, as previously Asia II 1 was also reported from Syria (a country from Middle East) because south Asian countries have been good trading partner with the countries of the Middle East.

## Supplementary Information

Supplementary Information 1.

Supplementary Information 2.
