# Supplementary material for: Dominance of Asia II 1 species of Bemisia tabaci in Pakistan and beyond
Source: Sci Rep. 2022 Jan 27;12:1528. doi: 10.1038/s41598-022-05612-1 (PMC8795192; doi:10.1038/s41598-022-05612-1)
Supplement: Supplementary file 1 — Supplementary Information 1. [file 41598_2022_5612_MOESM1_ESM.docx]

| **Sr. #** | **Countries** | **Species reported** |
| --- | --- | --- |
| **South Asia** | | |
| 1 | Pakistan | **Asia II 1**, MEAM1, Asia II 7, Asia 1, Asia II 5, Asia II 8, Pakistan, Pakistan 1 |
| 2 | India | Asia 1, **Asia II 1**, Asia II 8, Asia II 7, Asia II 5, MEAM1, China 3, Asia II 11, China 7, MEAMK |
| 3 | Bangladesh | Asia 1, Asia II 5, **Asia II 1**, Asia-II 10, China 3 |
| 4 | Nepal | Asia II 5, **Asia II 1**, Asia 1 |
| 5 | Afghanistan | Asia 1, Asia II 5 |
| **South East Asia** | | |
| 1 | Indonesia | Asia 1, Asia II 5, Asia II 6, Asia II 7, Asia II 9, Asia II 12, Australia, MEAM1 |
| 2 | Myanmar | Asia 1, Asia II 5 |
| 3 | Singapore | Asia1 |
| 4 | Cambodia | Asia 1, **Asia II 1** |
| 5 | Vietnam | Asia 1, **Asia II 1**, Asia II 6, MEAM1, Uganda 1 |
| 6 | Philippines | Asia 1, Asia II 6, Asia II 14, Asia II 15, Australia, MEAM1 |
| 7 | Thailand | Asia 1, **Asia II 1**, Asia II 10, Asia II 6, Uganda 1 |
| 8 | Malaysia | Asia 1, MED, Asia II 6, China 2, Asia II 7, China 1, Asia II 10 |
| **East Asia** | | |
| 1 | Japan | Asia 1, Asia II 13, Asia II 6, Japan 1, Japan 2, MEAM1, MEAM2, MED |
| 2 | South Korea | Japan 2, MEAM1, MEAMK, MED |
| 3 | Taiwan | Asia 1, **Asia II 1**, Asia II 13, Asia II 5, Asia II 6, Asia II 7, MEAM1, Sub Sahara Africa 2, MED |
| 4 | China | MED, MEAM1, Asia 1, China 1, Asia II 3, **Asia II 1**, Asia II 6, MEAMK, Asia-II 7, Asia II 9, China 3, China 5, MEAM2, China 2, Asia II 10, Asia II 2, Asia II 4, Asia V, China 4 |
| **Middle East** | | |
| 1 | Israel | MED, MEAM1, MEAMK |
| 2 | Turkey | Asia 1, MEAM1, MEAMK, MED |
| 3 | Cyprus | MED, MEAM1 |
| 4 | Egypt | MED, MEAM1, MEAMK |
| 5 | UAE | MEAM1 |
| 6 | Syria | Asia 1, **Asia II 1**, MEAM1, MED |
| 7 | Yemen | MEAM1 |
| 8 | Iran | MEAM1 |
| 9 | Jordan | MEAM1 |
| 10 | Kuwait | MEAM1 |
| 11 | Saudi Arabia | MEAM1 |
| 12 | Iraq | MEAM1, MEAM2 |
| 13 | Oman | MEAM1 |

**Supplementary Table 1.** Summary of the biotypes of B. tabaci identified from the countries of Asia and Middle East regions
